# Garlic and Resveratrol Attenuate Diabetic Complications, Loss of β-Cells, Pancreatic and Hepatic Oxidative Stress in Streptozotocin-Induced Diabetic Rats

**DOI:** 10.3389/fphar.2016.00360

**Published:** 2016-10-13

**Authors:** Gagandeep Kaur, Raju Padiya, Ramu Adela, Uday K. Putcha, G. S. Reddy, B. R. Reddy, K. P. Kumar, Sumana Chakravarty, Sanjay K. Banerjee

**Affiliations:** ^1^Division of Medicinal Chemistry and Pharmacology, Indian Institute of Chemical TechnologyHyderabad, India; ^2^Department of Biochemistry, Osmania UniversityHyderabad, India; ^3^Drug Discovery Research Center, Translational Health Science and Technology Institute, NCR Biotech Science ClusterFaridabad, India; ^4^Division of Pathology, National Institute of NutritionHyderabad, India; ^5^Division of Chemical Biology, Indian Institute of Chemical TechnologyHyderabad, India

**Keywords:** type-I diabetes, STZ, garlic, resveratrol, metformin, beta cell

## Abstract

The study was aimed at finding the effect of garlic and resveratrol on loss of β-cells and diabetic complication in streptozotocin (STZ)-induced Type-I diabetic rats. Rats were injected with single dose STZ (50 mg/kg, i.p.) for induction of type 1 diabetes (Dia) and compared with control group. Rats from third (Dia+Gar), fourth (Dia+Resv), and fifth (Dia+Met) groups were fed raw garlic homogenate (250 mg/kg/day), resveratrol (25 mg/kg/day), and metformin (500 mg/kg/day) orally, respectively, for a period of 4 weeks. Diabetic group had decreased serum insulin and hydrogen sulfide levels along with increased blood glucose and glycated hemoglobin, triglyceride, uric acid, and nitric oxide levels. Significant (*p* < 0.05) increase in pancreatic and hepatic TBARS, conjugated dienes, nitric oxide, and AGE level and significant (*p* < 0.05) decrease in SOD, catalase, H_2_S, GSH level were observed in diabetic group. Administration of garlic, resveratrol, and metformin significantly (*p* < 0.05) normalized most of the altered metabolic and oxidative stress parameters as well as histopathological changes. Administration of garlic, resveratrol, and metformin in diabetic rat decreases pancreatic β-cell damage and hepatic injury. Our data concluded that administration of garlic showed more promising effect in terms of reducing oxidative stress and pathological changes when compared to resveratrol and metformin groups.

## Introduction

Type-I diabetes mellitus (T1DM) is an autoimmune disease, which is found to be associated with the mass destruction of insulin-producing beta cells in pancreas (Trucco, 2005; Koulmanda, 2006). In T1DM, loss and degeneration of beta cells are associated with oxidative stress, i.e., production of reactive oxygen species (ROS) and sharp reduction of antioxidant defenses (Drews et al., 2010; Henriksen et al., 2011). Thus increased ROS level is responsible for decreased insulin secretion and increased beta cell damage (Evans et al., 2002; Rains and Jain, 2011). Oxidative stress in different organs increased due to various factors, i.e., increased glucose autoxidation, increased polyol pathway, increased advanced glycation end products, decreased antioxidant defenses, and increased ROS (Maritim et al., 2003; Al-Rawi, 2011).

Natural antioxidants derived from plants are good candidates to inhibit the formation of free radicals and thus are effective to prevent oxidative damage (Ashour et al., 2011). A good measure of scientific literature has shown the efficacy of plants and different nutritional agents to reduce diabetes and its complications (Alarcon-Aguilara et al., 1998). Plants and natural products are generally found less toxic and free from side effects than synthetic ones (Pari and Umamaheswari, 2000). Although insulin is the best therapy for Type-I diabetes and reduce blood glucose level, it possesses prominent side effects and often fails to alter the complications of diabetes (Jain et al., 2006). There are different mechanisms by which natural compounds show their efficacy in reducing blood glucose level. Among those mechanisms, enhancing insulin’s activity, inhibiting insulinase activity, improving β-cell mass, and increasing the regeneration of cells are a few of the promising ones (Shanmugasundaram et al., 1990).

Garlic (*Allium sativum*) under the family Liliaceae, is a well-known herb with medicinal value and has been used for both nutritional and medicinal purposes since ancient times (Thomson et al., 2007). Recent studies also reveal the beneficial effects of garlic or its preparations in combating various diseases. Hypo-lipidaemic, anti-atherosclerotic, anticoagulant, antidiabetic, antihypertensive, antimicrobial, anticancer, antidote, hepatoprotective, and immunomodulatory activities of garlic are now well- established from animal and human studies (Rajani Kanth et al., 2008). The salutary effects of garlic in Type-I and Type-II diabetes are also well established (Padiya et al., 2011). Besides its antidiabetic property, garlic could also be useful to reduce several diabetic complications due to its antioxidant property through scavenging ROS. The beneficial action of garlic may be due to the presence of several bioactive organosulfur compounds.

Resveratrol (trans-3, 5, 4′-trihydroxystilbene), another naturally occurring polyphenol phytoalexin compound with a strong antioxidant property, is found in grapes, peanuts, blueberries, and red wine. It has shown beneficial effects in a wide range of diseases like diabetes, inflammation, aging, and cancer (Pirola and Fro, 2008). Resveratrol is also observed to be effective in different animal models of diabetes such as streptozotocin, NOD mice and other genetic animal models (Baur et al., 2006; Su et al., 2006; Rivera et al., 2009; Palsamy and Subramanian, 2010; Lee et al., 2011). There is an extensive literature concerning the biological actions of resveratrol in reducing diabetic complications in many organs and tissue, including liver and pancreatic β-cells (Wu et al., 2012; Rouse et al., 2014; Bagul et al., 2015; Szkudelski and Szkudelska, 2015). However, more evidence on the antidiabetic activity of resveratrol along with its diverse biological action that directly or indirectly reduces diabetic complications in streptozotocin-induced animals is required.

Here, we investigated the antidiabetic effect of resveratrol and garlic in STZ-induced Type-I diabetic rats over 4 weeks experimental period. Previously, several research articles showed the beneficial effect of garlic and resveratrol in both type 1 and type 2 diabetic models and revealed diverse mechanisms for their antidiabetic effect. In the present study, we aimed to investigate whether resveratrol and garlic have a similar property to increase insulin secretion from beta cells as well as reducing tissue complication. We have tried to understand the disease complexity in a broader picture and integrated the pancreas, liver and serum parameters together to find the overall effect of garlic and resveratrol, and compared the effect with a standard drug, metformin.

## Materials and Methods

### Preparation of Garlic Homogenate

Fresh garlic (*Allium Sativum* L.) was purchased from a fixed shop in a local market in Hyderabad, India and authenticated by Dr. K MadhavaChetty, an ethnobotanist at Osmania University, Hyderabad, India. The specimen was deposited in the herbarium of Department of Botany, Osmania University and bears the voucher No. 0359. Garlic paste was made with grinder as described before (Banerjee et al., 2001). Fresh garlic homogenate was prepared every day before feeding to rat. The chemical composition of fresh raw garlic homogenate was determined previously using LC-MS (Padiya et al., 2014). The five major compounds present in garlic homogenates are γ-glutamyl-*S*-allyl-L-cysteine (Rt1.7 min, 291 m/z); Alliin (Rt 7.7 min, 178 m/z); *S*-allyl-L-cysteine (Rt 8.5 min, 162 m/z); Vinyldithiin (Rt 9.1 min, 145 m/z), and Allicin (Rt 11.9 min, 163 m/z) (Padiya et al., 2014).

### Animals and Treatment

All animal experiments were undertaken with the approval of Institutional Animal Ethical Committee of Indian Institute of Chemical Technology, Hyderabad. Male Sprague–Dawley rats (200–250 g) were purchased from the National Institute of Nutrition (NIN), Hyderabad, India. The animals were housed in BIOSAFE, an animal quarantine facility of Indian Institute of Chemical Technology, Hyderabad, India. The animal house was maintained at temperature 22 ± 2°C with relative humidity of 50 ± 15% and 12 h dark/light cycle. Animals were randomly divided into five groups (*n* = 8). The control group was fed normal diet. Diabetic rats were given a single dose streptozotocin (50 mg/kg, i.p) in freshly prepared citrate buffer to induce hyperglycemia. After 48 h of streptozotocin injection, blood glucose level of rats was measured. Rats with blood glucose levels more than 200 mg/dl were included in the diabetic group (Dia group). Control group rats (Con group) were administered equal volume of citrate buffer (i.p) to nullify its effect. The third group (Dia+Gar) diabetic rats were fed raw garlic homogenate (250 mg/kg) orally for a period of 4 weeks. The fourth (Dia+Resv) and fifth (Dia+Met) groups diabetic rats were fed resveratrol (25 mg/kg) and metformin (500 mg/kg) orally, respectively, for a period of 4 weeks. Garlic, resveratrol and metformin were administered orally to rats using oral gavage needle. Resveratrol was supplied by Kumar Organic Products Ltd., Bangalore, India. The dose of garlic, resveratrol and metformin was selected based on the previous literatures (Banerjee et al., 2001; Kim et al., 2006; Padiya et al., 2011; Padiya et al., 2014; Bagul et al., 2015). Resveratrol and metformin were prepared in 25% of DMSO. Control and Diabetic groups were treated with 25% DMSO to nullify the effects of DMSO.

### Estimation of Food Intake and Body Weight Gain

Throughout the experiment period, alterations of body weight and food intake in all groups were noted. The weight of each rat was recorded on day 0 and at weekly intervals. The quantity of food consumed by each group was recorded weekly. The food consumption per rat was calculated for all groups.

### Percentage of Mortality

As streptozotocin administration may cause acute hypoglycemia and death, percentage mortality was calculated by following formula: Mortality (%) = (Number of animals died/Total number of animals) × 100.

### Serum and Tissue Sample Collection

After 4 weeks of feeding and drug administration, the rats were anesthetized with ketamine (60 mg/kg) and xylazine (10 mg/kg), and sacrificed by cervical dislocation. Pancreas and liver tissues were collected and stored at -80^o^C for further biochemical evaluation. Blood was collected by retro-orbital plexus using small capillary tubes just before sacrificing the rats. Serum was separated by centrifugation at 5000 rpm (4°C) for 10 min and serum parameters were analyzed by auto blood analyzer. Both pancreas and liver tissues were homogenized with 10 times and 20 times volume, respectively, in ice cold 0.05 M potassium phosphate buffer (pH 7.4) and processed separately for different biochemical parameters (Sojitra et al., 2011).

### Biochemical Assays for Serum Parameters

Blood samples from all groups were analyzed at different time intervals to confirm the induction of diabetes and metabolic parameters. Serum glucose was determined after 2nd day and 1st week of Streptozotocin, while glycated hemoglobin, serum insulin, triglyceride levels, uric acid, nitric oxide, and H_2_S levels were determined after 4 weeks of streptozotocin.

### Estimation of Blood Glucose, Serum Uric Acid, Triglyceride, and Insulin

Blood glucose was measured directly from a small drop of blood using glucometer (One Touch Horizon, Singapore). Serum triglyceride and uric acid were measured using an auto blood analyzer as described (Bagul et al., 2012). Serum uric acid and triglyceride kits were obtained from Siemens, India. Estimation of serum insulin levels was done by ELISA kits (Mercodia, USA) (Padiya et al., 2011).

### Estimation of Nitric Oxide (NO) and Hydrogen Sulfide (H_2_S)

Nitric oxide was measured by a commercially available kit (Assay design, USA). Principle of assay is based on reduction of NO^3-^ into NO^2-^ using nitrate reductase. Azo dye was produced after diazotization of sulfanilic acid (Griess Reagent-1) with NO^2-^ and subsequent coupling with N-(1- napthyl)-ethylene diamine (Griess Reagent-2). The azo dye was measured calorimetrically at 540 nm. Serum NO level was expressed as μmol/L (Khatua et al., 2012).

Serum H_2_S concentration was measured by biochemical method as described by Cai et al. (2007) after some modifications. Briefly, 0.1 ml serum was added into solutions containing 0.125 ml of 1% zinc acetate and 0.15 ml distilled water. 0.067 ml of 20 mM N, *N*-dimethyl-phenylene diamine dihydrochloride in 7.2 M HCL was added into solution followed by addition of 0.067 ml of 30 mM FeCl3 in 1.2 M HCL. The absorbance of resulting solution was measured at a wavelength of 670 nm. The H_2_S concentration in a solution was calculated according to the standard curve of sodium hydrogen sulfide (NaHS: 3.12–400 μmol) and data were expressed as H_2_S concentration in μmol/L.

### Estimation of Glycated Hemoglobin (HbA1c)

Glycated hemoglobin was measured to confirm the chronic elevation of blood glucose level in diabetic rats. It was estimated using ion exchange micro-columns (Biosystem Ltd, Spain). After preparing the hemolysate according to the protocol, hemoglobin was retained by a cationic exchange resin. Glycated hemoglobin (HbA1c) was then specifically eluted after washing away the hemoglobin A1a and A1b fractions, and was estimated by spectrophotometric reading at 415 nm.

### Estimation of Pancreatic and Hepatic Oxidative Stress

For TBARS (a measure of MDA) estimation, whole tissue homogenate was used. Supernatant collected after centrifugation at 15,000 × *g* for 30 min at 4°C, was used for the estimation of conjugated dienes, NO, GSH, SOD, H_2_S, catalase, and AGE. Thio-barbituric acid reactive substances (TBARS) conjugated dienes, advanced glycation end products (AGE), and nitric oxide (NO) were measured as markers of lipid peroxidation, while reduced glutathione (GSH), superoxide dismutase (SOD), catalase (CAT), and H_2_S were estimated as levels of antioxidants. Chemicals that used for the estimation of different biochemical parameters were obtained from Sigma Inc., USA.

### TBARS, Conjugated Dienes, Nitric Oxide (NO), and Advanced Glycation End Product (AGE)

In pancreas and liver, the extent of lipid peroxidation was determined by measuring tissue TBARS content as described previously (Mahon et al., 1979). For estimating conjugated dienes, pancreas and liver tissues were treated with chloroform: methanol (2:1) followed by vigorous vortexing and centrifugation at 2000 rpm for 10 min after removing the upper layer. The lower chloroform layer was dried at room temperatures. The residue was dissolved in cyclohexane and absorbance was measured at 233 nm against a cyclohexane (standard 1 O.D. = 37.5 nmol) (Devasagayam et al., 2003). For estimating tissue NO, the supernatants from pancreas and liver homogenate were used and measured according to the method described earlier. For measurement of AGE, 200 μl supernatant from pancreas and liver homogenate was mixed with 500 μl of sodium phosphate buffer and incubated for 96 h (4 days) at a temperature of 50°C in a water bath. The fluorescence was measured by using emissions at 440 nm upon excitations at 370 nm (Munch et al., 1997; Banerjee et al., 2002).

### Catalase, SOD, GSH, and H_2_S

Catalase activity in pancreas and liver supernatant was determined by measuring the decomposition of hydrogen peroxide at 240 nm (Banerjee et al., 2002). Data were expressed as milliunits per microgram protein. SOD activities in pancreas and liver supernatant were determined according to the method provided by commercially available SOD assay kit (Fluka Analytical, Switzerland, catalog No. 19160). Superoxide anion produced by the activity of xanthine oxidase was inhibited by pancreatic and liver SOD. Data were expressed as % inhibition (Khatua et al., 2012). Glutathione (GSH) content in pancreas and liver supernatant was measured by biochemical assay using dithionitrobenzoic acid (DTNB) method (Banerjee et al., 2002). Data were expressed as μg/g wet tissue. H_2_S concentration was measured as described before. Data were expressed as H_2_S concentration in μmol/g tissue weight.

### Protein Estimation

Protein concentrations were measured using Bradford reagent according to the method described by Lowry et al. (1951).

### Histopathology

The pancreas and liver from each group (*n* = 2 per group) were fixed in 10% neutral buffered formalin for 48 h. The liver tissue was mounted on the section stage with the appropriate adhesive, 10 μm thin sections were cut on Oscillating Tissue Slicer (Model no. OTS-4500, Harvard Apparatus, USA) as described earlier (Bagul et al., 2012). Whereas for pancreas, tissue paraffin blocking was done and 5 μm thin sections were cut using regular rotary microtome. For both the tissues, only selected good sections were mounted on positively charged superfrost plus slides (Fisher Scientific, USA). Then sections were stained with hematoxylin and eosin (H and E), dehydrated with graded series of alcohol and mounted with DPX. The stained slides were observed using Axioplan Imaging System (MC200, Carl Zeiss Inclusive, Germany) and results were analyzed.

### Immunohistochemistry (IHC) of Pancreas to Stain Insulin Release

Immunohistochemistry (IHC) of pancreas was performed according to the method described previously (Chang and Kwon, 2000). Pancreas from each group was removed at the end of the experiment and stored in 10% formalin. Five micron thick paraffin blocks were sectioned and mounted on gelatinized slides coated with poly-L-lysine. Linked avidin biotin horseradish per-oxidase method (LABS-HRP) using mouse monoclonal IgG as primary antibody was used for detecting insulin containing beta cells. For this purpose, sections were deparaffinized, blocked with blocking agent and subsequently incubated with the primary antibody (Santa Cruz Catalog no. SC56418) or its negative reagent (non-immune guinea pig serum) at room temperature for 10 min. Sections were rinsed with three changes of PBS and then incubated with biotinylated secondary antibody. In the later stage, sections were incubated with streptavidin conjugated horseradish peroxidase. The peroxidase reaction was developed with 25 mg/dL deaminobenzidine in phosphate buffered saline with 0.025% hydrogen peroxide for 10 min at room temperature. Sections were examined by light microscope.

## Statistical Analysis

All values are expressed as mean ± SEM. Data were statistically analyzed by using one-way ANOVA for multiple group comparison, followed by Bonferroni-correction for group wise comparison. Significance was set at *P* < 0.05.

## Results

### Alteration of Food Intake and Body Weight Gain in Diabetic Rats after Garlic and Resveratrol Administration

**FIGURE 1:**
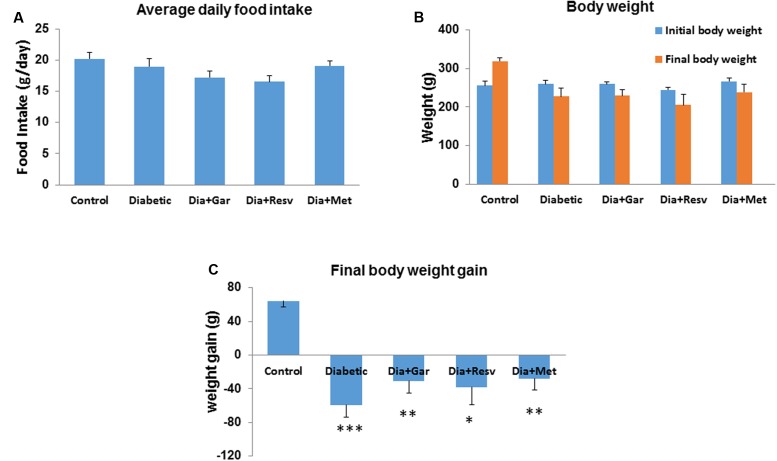
**Effect of garlic and resveratrol on food intake and body weight gain.** **(A)** Bar graph showing average daily food intake from first day to the end of 4 weeks. **(B)** Bar graph showing initial and final body weight of rats from all groups. **(C)** Bar graph showing body weight gain or loss from all groups during the experimental period. Data are shown as mean ± SEM, (*N* = 8) ^∗^*p* < 0.05, ^∗∗^*p* < 0.01, ^∗∗∗^*p* < 0.001 vs. Control.

There was no significant change of daily food intake between Control and Diabetic group. Similarly no significant change was observed in Diabetic groups after administration of garlic, resveratrol and metformin (Dia+Gar, Dia+Resv, and Dia+Met groups) (**Figure 1A**). Rats from Control group showed gain in body weight while those from Diabetic group showed loss of body weight. Administration of garlic, resveratrol, and metformin in Diabetic group (Dia+Gar, Dia+Resv, and Dia+Met groups) improved the body weight loss but not significant when compared to only Diabetic group (**Figures 1B,C**).

### Improvement of Percentage Mortality in Diabetic Rats after Garlic and Resveratrol Administration

Percentage mortality was calculated as per the formula mentioned earlier. There was no mortality found in Control, Dia+Gar, and Dia+Met groups. However, in case of Diabetic and Dia+Resv groups two and one animals died, respectively (**Table 1**).

**Table 1:** Percentage mortality after 4 weeks of experimental period.

Groups	Number of animal taken	Number of animal died	Percentage mortality
Control	8	0	0
Diabetic	8	2	25%
Dia+Gar	8	0	0
Dia+Resv	8	1	12.5%
Dia+Met	8	0	0


### Attenuation of Blood Glucose, Glycated Hemoglobin, and Insulin Levels after Garlic and Resveratrol Administration

**FIGURE 2:**
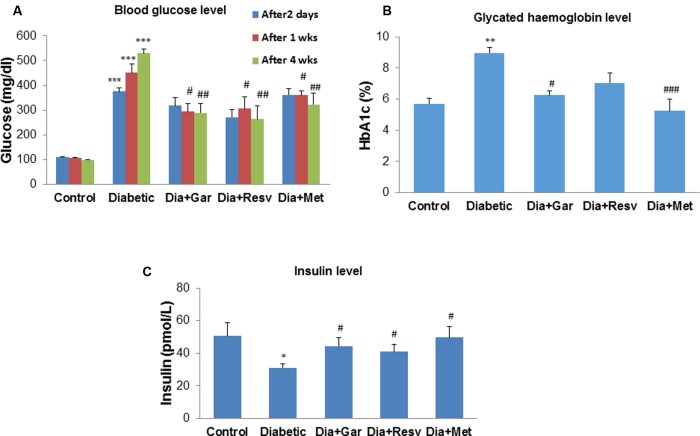
**Effect of garlic and resveratrol on blood glucose, glycated hemoglobin, and serum insulin levels **(A)** Blood glucose levels after 2 days, 1 and 4 weeks of STZ administration.** **(B)** Glycated hemoglobin levels in blood after 4 weeks of STZ administration **(C)** Serum insulin levels after 4 weeks of STZ. Data are shown as Mean ± SEM, (*N* = 8) ^∗∗∗^*p* < 0.001, ^∗∗^*p* < 0.01, ^∗^*p* < 0.05 vs. Control group; ^#^*p* < 0.05, ^##^*p* < 0.01, ^###^*p* < 0.001 vs. Diabetic group.

After 2 days, 1, 2, and 4 weeks, rats from the Diabetic group showed a significant (*p* < 0.05) increase in blood glucose levels compared to Control group. We have observed no attenuation of blood glucose levels in Dia+Gar, Dia+Resv, and Dia+Met groups after 2 days when compared to Dia group (**Figure 2A**). However, after 4 weeks, significant (*p* < 0.05) decrease was observed in all three different treatment groups. After 4 weeks, increased (*p* < 0.01) blood glycated hemoglobin level in Diabetic group was decreased significantly (*p* < 0.05) in Dia+Gar and Dia+Met groups but not in Dia+Resv group (**Figure 2B**). Similarly, decreased (*p* < 0.05) serum insulin level in Diabetic group was improved significantly (*p* < 0.05) after administration of garlic, resveratrol, and metformin (Dia+Gar, Dia+Resv, and Dia+Met) (**Figure 2C**).

### Improvement of Serum Triglyceride, Uric Acid, NO, and H_2_S Levels after Garlic and Resveratrol Administration

**FIGURE 3:**
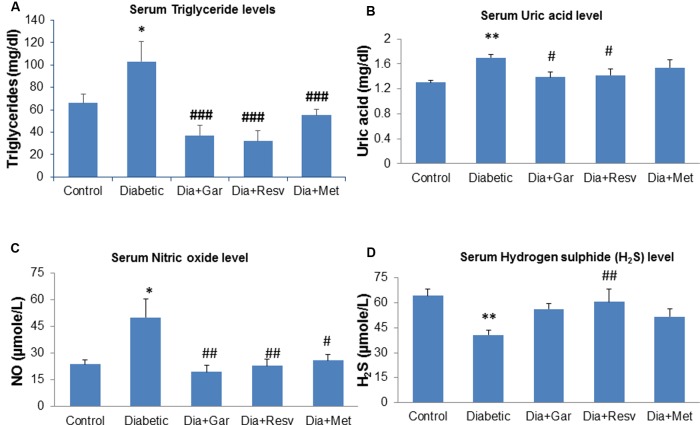
**Effect of garlic and resveratrol administration on serum metabolic parameters.** **(A)** Serum triglyceride levels after 4 weeks of STZ **(B)** Serum uric acid levels after 4 weeks of STZ **(C)** Serum nitric oxide levels after 4 weeks of STZ **(D)** Serum hydrogen sulfide levels after 4 weeks of STZ. Data are shown as Mean ± SEM, (*N* = 8) ^∗∗^*p* < 0.01, ^∗^*p* < 0.05 vs. Control group; ^#^*p* < 0.05, ^##^*p* < 0.01, ^###^*p* < 0.001 vs. Diabetic group.

After 4 weeks of streptozotocin administration, rats of Diabetic group showed a significant (*p* < 0.05) increase in serum triglyceride and nitric oxide level when compared to Control group. However, a significant (*p* < 0.001) decrease in serum triglyceride as well as nitric oxide level was observed after administration of garlic, resveratrol, and metformin (Dia+Gar, Dia+Resv, Dia+Met groups) compared to Diabetic group (**Figures 3A,C**). Increased (*p* < 0.001) serum uric acid level in Diabetic group was decreased significantly (*p* < 0.05) both in Dia+Gar and Dia+Resv groups but not in Dia+Met group (**Figure 3B**). Decreased (*p* < 0.001) serum H_2_S level in Diabetic group was increased significantly (*p* < 0.001) in Dia+Resv group but not in Dia+Gar and Dia+Met groups (**Figure 3D**).

### Attenuation of Pancreatic TBARS, Conjugated Dienes, NO, and AGE Levels after Garlic and Resveratrol Administration

**FIGURE 4:**
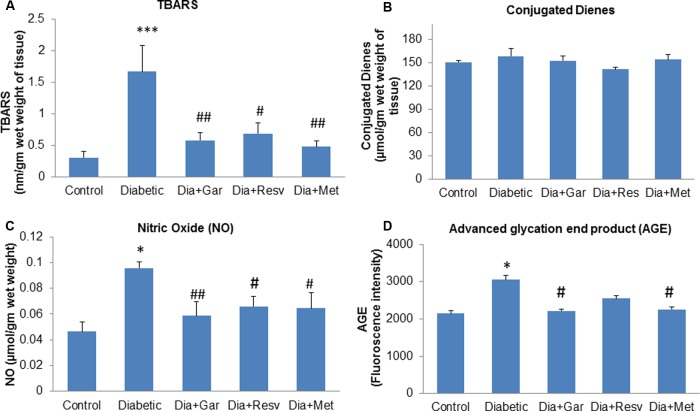
**Effect of garlic and resveratrol administration on oxidative stress parameters in pancreas.** **(A)** TBARS levels in pancreas after 4 weeks of STZ **(B)** Conjugated dienes levels in pancreas after 4 weeks of STZ **(C)** Nitric oxide levels in pancreas after 4 weeks of STZ **(D)** AGE levels in pancreas after 4 weeks of STZ. Data are shown as Mean ± SEM, (*N* = 5–8) ^∗^*p* < 0.05, ^∗∗∗^*p* < 0.001 vs. Control group; ^#^*p* < 0.05, ^##^*p* < 0.01 vs. Diabetic group.

Pancreatic TBARS levels were significantly (*p* < 0.001) increased in Diabetic group when compared with Control group. However, a significant (*p* < 0.05) decrease in pancreatic TBARS levels was observed after administration of garlic, resveratrol, and metformin (Dia+Gar, Dia+Resv, and Dia+Met groups) compared to Diabetic group (**Figure 4A**). Pancreatic Conjugated dienes did not show any significant change in Diabetic group when compared to Control group. Similarly no significant change was observed in Dia+Gar, Dia+Resv, and Dia+Met group when compared to Diabetic group (**Figure 4B**). Pancreatic nitric oxide levels were significantly (*p* < 0.05) increased in Diabetic group when compared with Control group. However, a significant (*p* < 0.05) decrease in pancreatic nitric oxide levels was observed in Dia+Gar and Dia+Met but not in Dia+Resv group when compared to Diabetic group (**Figure 4C**). Pancreatic AGE levels were significantly (*p* < 0.05) increased in Diabetic group when compared to control group. However, there was no significant change in pancreatic AGE in Dia+Gar, Dia+Resv, Dia+Met groups when compared to Diabetic group (**Figure 4D**).

### Normalization of Pancreatic Catalase, H_2_S, SOD, and GSH Levels after Garlic and Resveratrol Administration

**FIGURE 5:**
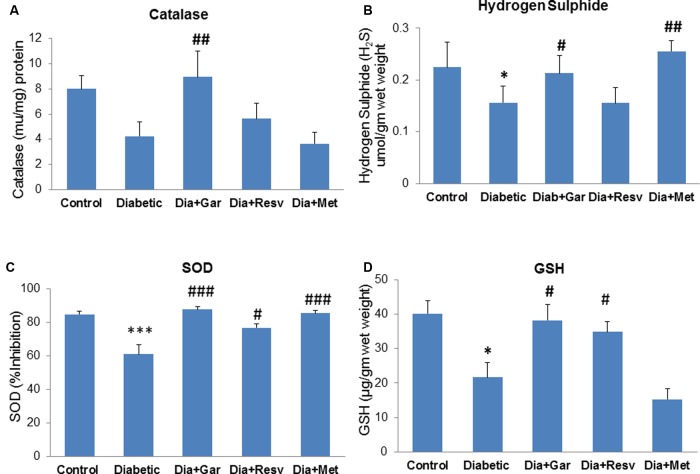
**Effect of garlic and resveratrol administration on pancreatic catalase, hydrogen sulfide, SOD, and GSH.** **(A)** Catalase activity in pancreas after 4 weeks of STZ **(B)** Hydrogen sulfide levels in pancreas after 4 weeks of STZ **(C)** SOD activity in pancreas after 4 weeks of STZ **(D)** GSH levels in pancreas after 4 weeks of STZ. Data are shown as Mean ± SEM, (*N* = 5–8) ^∗^*p* < 0.05, ^∗∗∗^*p* < 0.001 vs. Control group; ^#^*p* < 0.05, ^##^*p* < 0.01, ^###^*p* < 0.001 vs. Diabetic group.

There was a decrease but no significant change in pancreatic catalase activity was observed in Diabetic group compared to Control group. However, administration of only garlic (Dia+Gar group) significantly (*p* < 0.01) increased pancreatic catalase activity but no change was observed in Dia+Resv and Dia+Met groups compared to Diabetic group (**Figure 5A**). Pancreatic H_2_S levels were significantly (*p* < 0.05) decreased in Diabetic group when compared to Control group. However, Dia+Gar and Dia+Met showed (*p* < 0.05) significant increase in pancreatic H_2_S levels but no change was observed in Dia+Resv group compared to Diabetic group (**Figure 5B**). Pancreatic SOD activity was significantly (*p* < 0.001) reduced in Diabetic group. However, the SOD activity in diabetic rat was significantly (*p* < 0.05) increased in Dia+Gar, Dia+Resv, and Dia+Met groups (**Figure 5C**). Pancreatic GSH level was significantly (*p* < 0.05) reduced in Diabetic group when compared to Control group. However, administration of garlic and resveratrol significantly (*p* < 0.05) increased pancreatic GSH level but no significant change was observed after metformin administration (Dia+Resv group) compared to Diabetic group (**Figure 5D**).

### Attenuation of Hepatic TBARS, Conjugated Dienes, NO, and AGE Levels after Garlic and Resveratrol Administration

**FIGURE 6:**
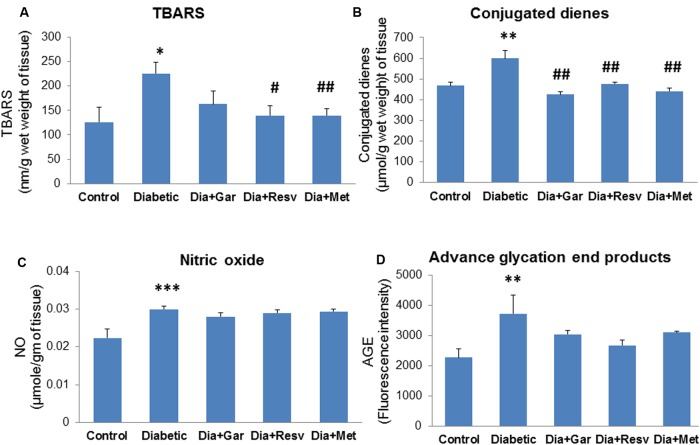
**Effect of garlic and resveratrol administration on oxidative stress parameters in liver.** **(A)** Hepatic TBARS levels after 4 weeks of STZ **(B)** Hepatic conjugated dienes levels after 4 weeks of STZ **(C)** Hepatic nitric oxide levels after 4 weeks of STZ **(D)** Hepatic AGE levels 4 weeks of STZ. Data are shown as Mean ± SEM, (*N* = 5–8) ^∗^*p* < 0.05, ^∗∗^*p* < 0.01, ^∗∗∗^*p* < 0.01 vs. Control group; ^#^*p* < 0.05, ^##^*p* < 0.01 vs. Diabetic group.

Hepatic TBARS levels were significantly (*p* < 0.05) increased in Diabetic group compared to Control group. Administration of resveratrol (Dia+Resv group) and metformin (Dia+Met group) but not garlic (Dia+Gar group) showed significant (*p* < 0.05) reduction of hepatic TBARS levels compared to Diabetic group (**Figure 6A**). Increased (*p* < 0.01) hepatic conjugated dienes level in Diabetic group was significantly (*p* < 0.01) decreased after administration of garlic, resveratrol, and metformin (Dia+Gar, Dia+Resv, and Dia+Met groups) (**Figure 6B**). Hepatic NO and AGE levels were significantly (*p* < 0.01) increased in Diabetic group when compared to Control group. However, these increased NO and AGE levels in Diabetic group were not decreased after administration of garlic, resveratrol, and metformin (Dia+Gar, Dia+Resv, and Dia+Met groups) (**Figures 6C,D**).

### Normalization of Hepatic Catalase, H_2_S, SOD, and GSH Levels after Garlic and Resveratrol Administration

**FIGURE 7:**
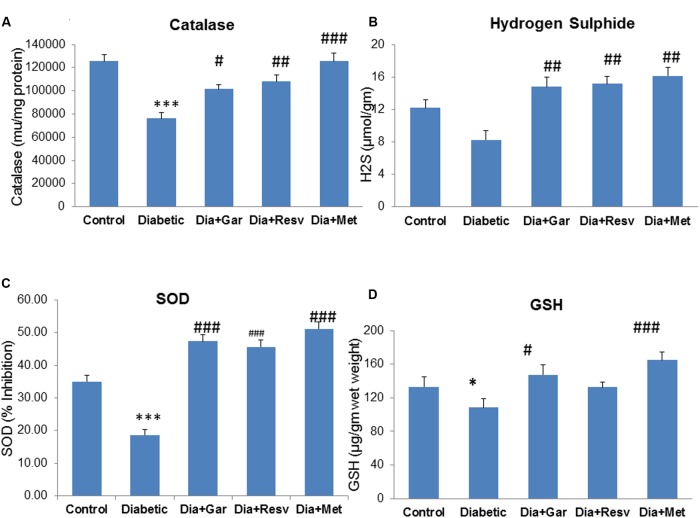
**Effect of garlic and resveratrol administration on hepatic catalase, hydrogen sulfide, SOD and GSH.** **(A)** Hepatic catalase activity after 4 weeks of STZ **(B)** Hepatic hydrogen sulfide levels after 4 weeks of STZ **(C)** Hepatic SOD activity after 4 weeks of STZ **(D)** Hepatic GSH levels after 4 weeks of STZ. Data are shown as Mean ± SEM (*N* = 5–8) ^∗^*p* < 0.05 vs. control, ^∗∗∗^*p* < 0.001 vs. Control group; ^#^*p* < 0.05, ^##^*p* < 0.01, ^###^*p* < 0.001 vs. Diabetic group.

Hepatic catalase activity was significantly (*p* < 0.001) decreased in Diabetic group compared to Control group. However, administration of garlic, resveratrol, metformin (Dia+Gar, Dia+Resv, and Dia+Met groups) showed significant (*p* < 0.05) increase in haptic catalase activity when compared to Diabetic group (**Figure 7A**). There was a decrease but no significant change in hepatic H_2_S levels was observed in Diabetic group compared to Control group. However, administration of garlic, resveratrol and metformin (Dia+Gar, Dia+Resv, and Dia+Met groups) showed significant (*p* < 0.01) increase in hepatic H_2_S levels compared to Diabetic group (**Figure 7B**). Hepatic SOD activity decreased significantly (*p* < 0.001) in Diabetic group compared to Control group. However, administration of garlic, resveratrol and metformin (Dia+Gar, Dia+Resv, and Dia+Met groups) showed significant (*p* < 0.001) increase in SOD activity compared to Diabetic group (**Figure 7C**). Hepatic GSH level was decreased significantly (*p* < 0.05) in Diabetic group compared to Control group. However, administration of garlic (Dia+Gar group) and metformin (Dia+Met group) but not resveratrol (Dia+Resv group) significantly (*p* < 0.05) increased the hepatic GSH levels (**Figure 7D**).

### Improvement of Pancreatic Damage in Diabetic Rats after Garlic and Resveratrol Administration

**FIGURE 8:**
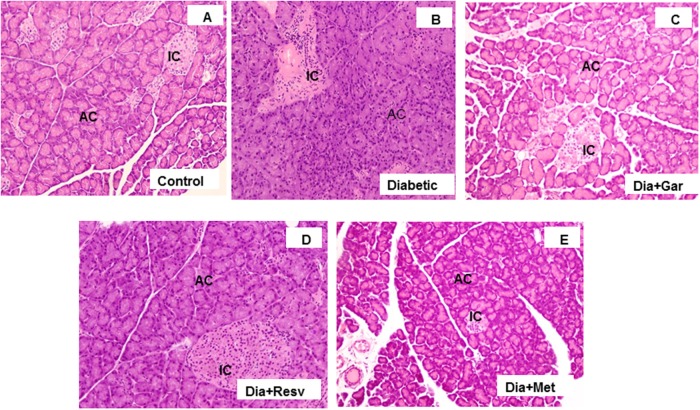
**Photomicrographs of rat pancreas, H&E, 200× Photomicrograph of control pancreas **(A)**: illustrating Islets cell (IC) were in normal proportion and embedded within the acinar cells (AC).** Pancreatic section of diabetic rat **(B)** showing decrease in number of islet cells and ACs. The Pancreatic sections from diabetic animals treated with both garlic **(C)** and resveratrol **(D)** showed normal proportion of acinar and islets cells. Pancreatic section of diabetic rat treated with metformin **(E)** showed smaller volume of islets cells compared to Control group.

Histopathological changes in pancreases were observed after Hematoxylin and Eosine (H&E) staining in all groups of animals (**Figure 8**). In Control group, acinar and beta cells were present in pancreas in their normal proportions. The acinar cells (ACs), which stained strongly, were arranged in lobules with prominent nuclei. The islet cells were seen embedded within the ACs and surrounded by a fine capsule (**Figure 8A**). In Diabetic group, although the ACs around the islets were present in normal proportion, the arrangement of cells was not classical rather demarcation of islet cells. There was a decrease in islet cells number, presence of fibrosis and disarrangement of cells in diabetic pancreas (**Figure 8B**). After administration of garlic (Dia+Gar group) and resveratrol (Dia+Resv), the ACs and the islets cells were observed in normal proportion (**Figures 8C,D**). After administration of metformin (Dia+Met), the ACs were showed in normal proportion and the islets cells were smaller in volume as compared with control (**Figure 8E**).

### Increased Insulin Staining in Beta Cells of Diabetic Pancreas after Garlic and Resveratrol Administration

**FIGURE 9:**
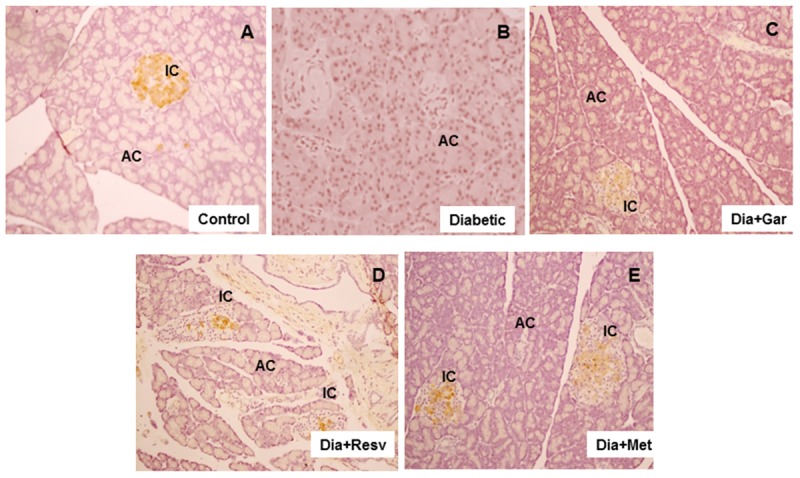
**Photomicrographs of rat pancreas after insulin staining, 200× **(A)** Photomicrograph of pancreas in Control group after immunostaining with insulin antibody.** Pancreatic islets showing normal insulin staining (brown). **(B)** Decrease or no brown staining of insulin in islets of pancreas in Diabetic group. **(C)** Improvement of brown insulin staining in islets of pancreas of diabetic rats after administration of garlic. **(D)** Improvement of brown insulin staining in islets of pancreas in diabetic rats after administration of resveratrol. **(E)** Improvement of insulin staining in islets of pancreas in diabetic rats after administration of metformin.

We looked the insulin secretion by staining method in pancreatic beta cells from each group. Pancreatic islet cells showed insulin staining (brown) in Control group and treated groups. However, beta cells in Diabetic group showed lower or no brown stain (**Figure 9**). In Control group, pancreas showed the typical component of beta cells occupying most of the islet cells. The ACs were arranged in lobules with prominent nuclei. The 27.53% insulin positive islets cells were present in Control group (**Figure 9A**). In Diabetic group, destruction of islet cells and ACs was observed. No or very less insulin positive islet cells with yellow stains were present (**Figure 9B**). After administration of garlic, 21% insulin positive islets cells were present in the pancreas of Dia+Gar group. The ACs were arranged in lobules same as Control (**Figure 9C**). After administration of resveratrol, 11% insulin positive islets cells were present (Dia+Resv group). The ACs were arranged in lobules surrounded by the islet cells (**Figure 9D**). After administration of metformin, the 10% positive islets cells were present in the pancreas of Dia+Met group (**Figure 9E**).

### Improvement of Hepatic Damage in Diabetic Rats after Garlic and Resveratrol Administration

**FIGURE 10:**
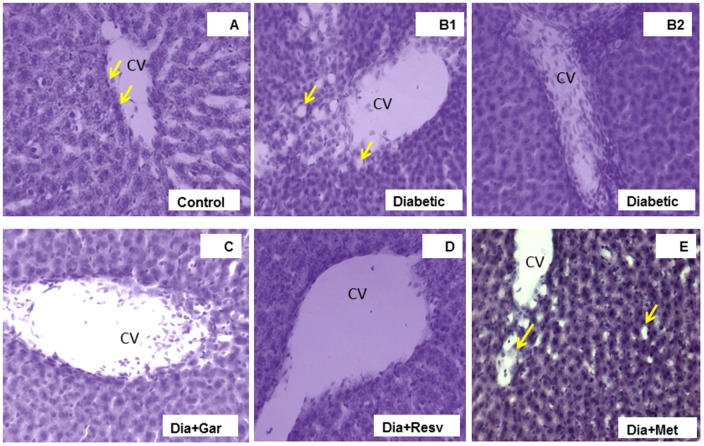
**Photomicrographs of rat liver, H&E, 20× Liver section of control rat **(A)** illustrating central vein (CV) with endothelial lining (arrows), hepatocytes and sinusoidal spaces with Kupffer cells.** Liver section of diabetic rat **(B1,2)** showing loss of the normal architecture due to distended central veins in different areas with lots of leucocytic infiltrations into the vein and in between vacuolations (yellow arrows) as damage hallmarks. The liver sections from diabetic animals treated with garlic **(C)**, resveratrol **(D),** or metformin **(E)** showed overall remarkable recovery changes toward normal histology with few exceptions **(D)**. Note still remains of few leucocytic infiltrations to CV in garlic treated group **(C)**, and slightly more vacuole spaces in metformin treated diabetic animals (arrows in **E**) than control.

The H&E stained liver sections of control rats showed the normal hepatic structure made up of hepatic lobules. Each lobule is made up of radiating plates, strands of cells forming a network around a central vein (**Figure 10A**). The liver strands were altering with narrow sinusoids, endothelial cells and Kupffer cells. In contrast, liver histology of Type-I diabetic rats (Diabetic group) was found to be degenerative. In some lobules, there were remarkable losses of normal architecture such as disarrangement of hepatocytes, distended central vein (**Figures 10B1,B2**) in different areas with lots of leucocytic infiltrations into the vein and in between vacuolations (yellow arrows). A number of cells were present with pycnotic appearance too. Interestingly, the liver sections from Type-I diabetic rats treated with either garlic (Dia+Gar group) (**Figure 10C**) and resveratrol (Dia+Resv group) (**Figure 10D**) were seem to be better recovered than that of metformin treated group (Dia+Met group) (**Figure 10E**). However, central veins were found to be a little dilated in garlic and resveratrol groups than normal but cells as such were found to be healthy. On the other hand, the liver histology of metformin treated T1D rats did not show a healthy appearance as many nucleuses were identified as pycnotic. More vacuole spaces were present in metformin treated diabetic animals (yellow arrows in E) than control.

## Discussion

Diabetes mellitus is a complex metabolic disorder, which leads to a series of biochemical and cellular events including oxidative stress, alterations of genes and protein expression, beta cell dysfunctions and hepatic cell injury. Since it difficult for a single drug to take care of all the events mentioned, it is legitimate to find an effective nutritional therapy, which can control most of the above detrimental events associated with this metabolic disease. Therefore, the present study was designed to find suitable nutritional and pharmacological agents, which can attenuate diabetes as well as oxidative stress and pathological changes in the pancreas and liver of diabetic rats. With this idea, we investigated the role of garlic and resveratrol in attenuating diabetes and its complication in Type-I diabetic rats.

Streptozotocin-induced diabetes is well-established model in rats. In the present study, we induced diabetes by single intra-peritoneal injection of 50 mg/kg body weight of STZ. Significant increase in blood glucose level was observed after 2 days, 1, 2, and 4 weeks of STZ administration. Induction of Type-I diabetes was associated with increased blood glycated hemoglobin level, decreased serum insulin level, decreased body weight and increased mortality rate as reported earlier (Su et al., 2006; Palsamy and Subramanian, 2010). Reduction of body weight is very common in Type-I diabetic patients. Similar to human, we also observed significant reduction of body weight in STZ-treated rats. Increased plasma glucose and glycated hemoglobin, decreased plasma insulin level and weight loss that occurred in STZ-induced diabetic rats were attenuated by garlic, resveratrol, and metformin. In addition, we observed 25% mortality in diabetic group after 4 weeks of STZ-administration. Resveratrol treatment reduced the mortality from 25 to 12.5%. However, garlic and metformin treatments had no mortality after 4 weeks of STZ-administration. Several biochemical parameters like serum triglyceride and uric acid level were also increased in Type-I diabetic rats. Our data is very similar to others that published previously (Su et al., 2006; Noriega-Cisneros et al., 2012). Administration of both garlic and resveratrol reduced serum triglyceride and uric acid level in comparison to standard drug metformin. Although garlic, resveratrol and metformin showed improvement in metabolic parameters, garlic showed greater improvement of insulin release compared to resveratrol and metformin as shown by IHC study. Our data also indicated that garlic has more beneficial effect than resveratrol in terms of mortality. The beneficial effect of garlic is due to the presence of allicin-type compounds or sulfur compounds [di (2-propenyl) disulfide and 2-propenyl propyl disulfide, respectively] (Chang and Johnson, 1980; Augusti, 1996). However, the present study clearly indicated that the increased insulin release from pancreatic beta cells might be responsible for improvement of diabetes after garlic and resveratrol treatment.

The overall mechanism might be mediated through the increase in pancreatic secretion of insulin from the β-cells or through the release of bound insulin as reported by Jain and Vyas (1975). Hypoglycemic activity of garlic may also be due to the inhibition of hepatic glucose production and/or stimulation of glucose utilization by peripheral tissues, especially muscle, and adipose tissues (Eddouks et al., 2005).

Diabetic complication in STZ-rats was also investigated by alteration of two important gaseous signaling molecules, NO and H_2_S. Both the molecules are thought to be very important in playing crucial role to regulate a wide range of physiological and pathological processes (Mancardi et al., 2011; Mattapally and Banerjee, 2011). Increased serum levels of NO (Adela et al., 2015) and decreased levels of H_2_S (Jain et al., 2010) have been reported in diabetic patients. Interestingly, we also found higher serum NO levels and lower H_2_S levels in diabetic rats. Increased NO and decreased H_2_S levels in STZ diabetic rats can also increase oxidative damage through formation of peroxynitrate (Pacher et al., 2011). Administration of garlic, resveratrol and metformin normalized both gaseous molecules in diabetic rats. Improvement of NO and H_2_S levels might be due to reduction of oxidative stress after treatment as observed in the present study.

Oxidative stress plays a major role in the development and complications of Type-I diabetes. Oxidative stress generally induces tissue injuries, damage of cellular organelles and cell death through production of ROS, reduction in endogenous antioxidants and increase in lipid peroxidation (Esterbauer and Cheeseman, 1990). In the present study, increased oxidative stress was evidenced by elevation of pancreatic and hepatic TBARS and conjugated dienes levels. Under hyperglycemia, production of various reducing sugars, such as glucose-6-phosphate and fructose increases through glycolysis and polyol pathways (Watford, 2002). During this process, ROS may be produced and cause tissue damage. In the present study, we observed significant increase in advanced glycation end product (AGE) in both liver and pancreas. Advanced glycation end products produced due to hyperglycemia can also interact with specific receptors on membranes to generate oxidants (Wautier et al., 1994). In the present study, administrations of garlic, resveratrol and metformin reduced the pancreatic and hepatic oxidative stress as observed by decreased TBARS and conjugated dienes levels. Reduction in pancreatic and hepatic AGE levels was also observed after administration of garlic, resveratrol and metformin.

STZ is a nitric oxide (NO) donor and NO has been found to destroy the pancreatic islet cells. It was proposed that NO molecule contributes to STZ-induced DNA damage. Lowering of NO concentration in pancreatic islet cells by inhibition of the inducible form of nitric oxide synthase partially counteracted STZ-induced DNA cleavage (Szkudelski, 2001). Similar to other studies, we found increase in NO levels in STZ-treated pancreas and liver tissues. Increased level of NO may be responsible for peroxynitrite production and cell damage. However, garlic, resveratrol and metformin treatment reduced the increased NO production. Reduction of NO levels might be responsible for reduced oxidative stress and β-cell damage. We also investigated the alteration of another gaseous signaling molecule, hydrogen sulfide (H_2_S) in the pancreas and liver. H_2_S has been shown to be protective in different cells against oxidative stress. We observed significant reduction of H_2_S levels in STZ-treated pancreas and liver. However, garlic, resveratrol, and metformin treatment significantly increased the pancreatic and hepatic H_2_S levels.

Antioxidants form an important part of a cell’s defense mechanism against free radical damage. Antioxidant enzymes, in particular, constitute a major part of this defense mechanism. SOD detoxifies peroxide radicals, giving rise to hydrogen peroxide (H_2_O_2_), and is the only known enzyme that uses free radicals as a substrate. Increased H_2_O_2_ production during oxidative stress can be neutralized by catalase and glutathione (GSH; Sullivan-Gunn and Lewandowski, 2013). Similar to other studies (Kumarappan et al., 2012; Singh et al., 2013), we have also observed significant reduction in SOD, catalase and GSH along with increase in TBARS and conjugated dienes in the STZ-treated pancreas and liver. The decreased levels of catalase, SOD and GSH along with increased levels of TBARS and conjugated dienes were normalized after administration of garlic, resveratrol and metformin.

To verify the pancreatic beta cell damage and hepatic injury, we performed pancreatic insulin immunostaining assay and histopathology study. Liver histology of Type-I diabetic rats found extensive hepatic injury, which was improved after garlic and resveratrol administration. Similarly, the histological abnormalities caused by diabetes were improved after treatment with garlic, resveratrol and metformin. However, histologically garlic protected pancreatic β-cell integrity more significantly than resveratrol and metformin. The IHC study with pancreatic islet cells showed higher insulin stain (brown) in control group. However, STZ-treated diabetic group showed very low or no brown stain in pancreatic beta cells. Although, all three treated groups increased the insulin staining in the STZ treated pancreas, garlic group showed more improvement than resveratrol and metformin.

The present study revealed that both resveratrol and garlic have similar property to increase insulin secretion from beta cells. This is reflected by the increase in serum insulin level as well as enhanced insulin staining in beta cells after resveratrol and garlic administration in diabetic rats. In conclusion, our data confirmed that garlic is superior in terms of normalizing serum parameters and oxidative stress in liver and pancreas, and attenuating β-cell destruction in type-1 diabetic rats.

## Author Contributions

GK, RP, SC, and SB conceived and designed the experiments; GK, RP, UP, GR, BR, KK, and RA performed the experiments; GK, RP, UP, GR, BR, KK, and RA, and SC analyzed the data; GK, RA, and SB wrote the paper.

## Conflict of Interest Statement

The authors declare that the research was conducted in the absence of any commercial or financial relationships that could be construed as a potential conflict of interest.
